# Colorimetric Determination of Sulfoxy Radicals and Sulfoxy Radical Scavenging-Based Antioxidant Activity

**DOI:** 10.1021/acsomega.3c03194

**Published:** 2023-09-27

**Authors:** Çiğdem Aşilioğlu, Seda Uzunboy, Sema Demirci-Çekiç, Reşat Apak

**Affiliations:** † Department of Chemistry, Institute of Graduate Studies, Istanbul University-Cerrahpaşa, Avcilar, Istanbul 34320, Turkey; ‡ Department of Chemistry, Faculty of Engineering, Istanbul University-Cerrahpasa, Avcilar, Istanbul 34320, Turkey; § Turkish Academy of Sciences (TUBA), Vedat Dalokay St. No. 112, Cankaya, Ankara 06670, Turkey

## Abstract

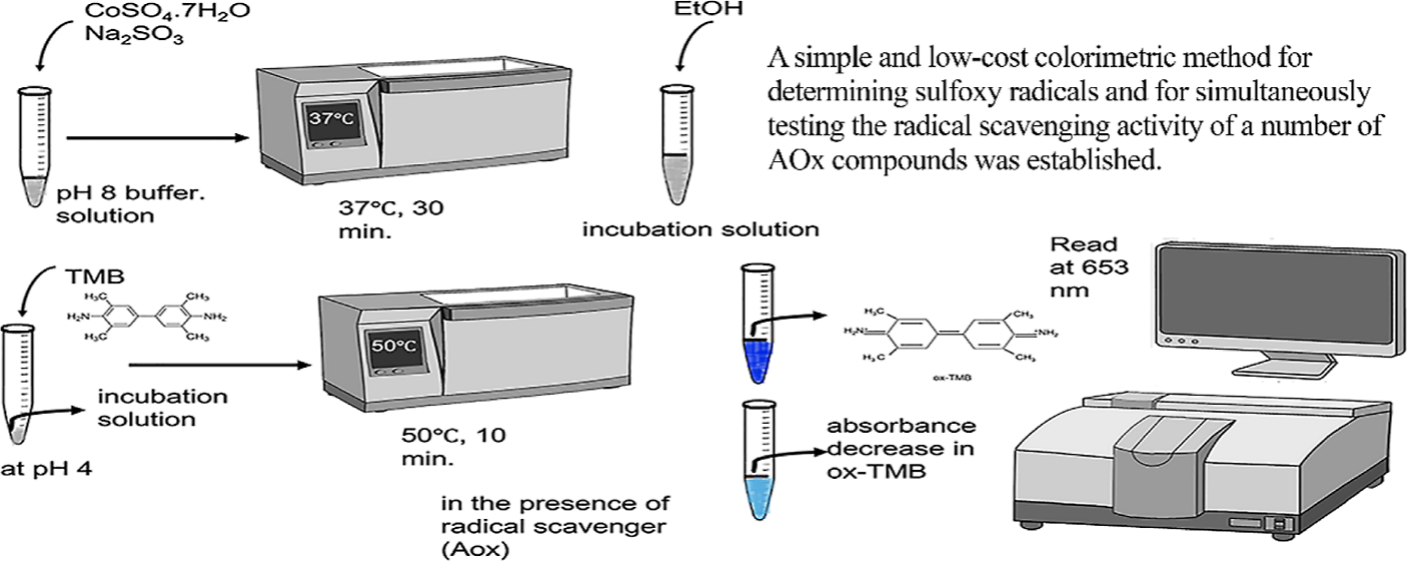

Sulfoxy radicals (SORs) are oxygen- and sulfur-containing species such as SO_3_^•–^, SO_4_^•–^, and SO_5_^•–^. They can be physiologically generated by S(IV) autoxidation with transition metal catalysis. Due to their harmful effects, the detection of both SORs and their scavengers are important. Here, a simple and cost-effective method for the determination of SORs and the scavenging activity of different antioxidant compounds was proposed. A SOR was selectively generated by combining CoSO_4_·7H_2_O with Na_2_SO_3_. To detect SOR species as a whole, 3,3′,5,5′-tetramethylbenzidine (TMB) was used as the chromogenic reagent, where SOR generated in the medium caused the formation of a blue-colored diimine from TMB. Additionally, the SOR scavenging effects of a number of antioxidant compounds (AOx) belonging to different classes were investigated, among which catechin derivatives were the most effective scavengers. The obtained results were compared with those of a reference rhodamine B decolorization assay. The radical scavenging effects of the tested AOx were ranked by both assays and then compared using the Spearman statistical test to yield a very strong correlation between the two rankings. The method was applied to real samples such as catechin-rich tea, that is, white, black, and green tea, among which white tea was determined as the most effective SOR scavenger.

## 1 Introduction

A group of radical species consisting of sulfur and oxygen (SO_2_^•^, SO_3_^•–^, SO_4_^•–^, O_3_SOO^•−^, etc.) is named as sulfoxy radicals (SORs). In the human body, enzymatic conversion of sulfite may cause generation of SORs.^1^ In the environment we live in, there are possible external sulfite sources that may cause SOR formation in the human body, such as SO_2_ gas in polluted air, foodstuffs containing sulfite as preservative (e.g., sulfited-dried apricots and wine),^2^ and sulfur-containing drugs (e.g., *N*-acetylcysteine (NAC)). Additionally, the normal metabolism of sulfur-containing amino acids can be mentioned as another sulfite source.^3^ During autoxidation in the presence of transition metal ions, sulfite species are oxidized in a pH-dependent manner to give SO_4_^2–^ as the final product. The reaction proceeds through several steps involving generation of different SORs,^4^ which may cause harm to cell membranes and biomacromolecules such as proteins and DNA. Oxidation of sulfite can be catalyzed by transition metal ions, particularly of cobalt.^5^ It was proposed that Co(II), Cu(II), and Mn(II) can cause site-specific DNA damage in the presence of S(IV) due to SO_4_^•–^ formation; the authors reported the order of activity of the tested transition metal ions on sulfite-induced DNA damage as Co(II) > Cu(II) > Mn(II) > Fe(III). Another finding of the researchers was that the damage could be inhibited by primary and secondary alcohols but not SOD, catalase, and *tert*-butanol.^6^ Some epidemiological studies showed a relation between development of lung cancer and SO_2_ exposure.^7^ Although the mechanism of sulfite toxicity in the body is not fully understood, it may involve the generation of SORs.^3^

One of the essential transition elements in the body is cobalt, which takes part in the formation of vitamin B12 (or its synthetic compound, cyanocobalamin). Although Co in vitamin B12 is tightly bound to a corrin ring, some ionic cobalt inevitably enters the body. Another extensively consumed protein source is yeast, containing Co-substituted metalloproteins.^8^ A number of foodstuffs such as chocolate, coffee, nuts, and vegetables with green leaves also contain Co in different amounts. Finally, workers in certain industries such as metal, construction, e-waste recycling, pigment production, and paint may be more exposed to cobalt than the standard population.^9^

There are studies related to the formation of sulfate radicals (SO_4_^•–^) as a leading SOR, emerging from the reaction between a sulfur-containing molecule and a transition metal ion. As a common point in these studies, it has been pointed that Co^2+^ is the most effective transition metal for SO_4_^•–^ generation among tested metal ions (such as Ag^+^, Ce^3+^, Co^2+^, Fe^3+^, Fe^2+^, Mn^2+^, Ni^2+^, Ru^3+^, and V^3+^).^10,11^

In this study, we aimed to investigate an effective way to produce SORs and offer a simple, low-cost colorimetric quantification method for SORs and their scavengers. In spite of the importance of SORs for human health, there are very limited studies on their colorimetric determination since basic attention is focused on other reactive oxygen species (ROS). Moreover, SOR scavengers, which may reduce the effectiveness of certain advanced oxidation processes in water treatment, were not studied in detail. Recently, Uzunboy et al. presented a novel spectrophotometric method for detecting sulfate radicals (SO_4_^•–^) generated by Cr^3+^/K_2_S_2_O_8_ and investigated the effects of certain radical scavengers.^12^ The presented work was not intended merely for the SOR assay, but different AOx compounds belonging to different subclasses were tested and ranked according to their SOR scavenging activities. The obtained results were compared to those of the rhodamine B decolorization assay as a reference. The SOR scavenging activity orders obtained by both methods were compared according to Spearman rank correlation coefficient statistics.^13^ The AOx rank results showed a very strong relationship between the two scavenging activity orders. Finally, green, white, and black tea infusions were used as real samples for testing the radical scavenging efficacies determined by the proposed and reference methods. To the best of our knowledge, there has been no study either on the determination of SORs produced from a combination of Co(II) and sulfite or on a comprehensive examination of SOR scavenging efficacies of different AOx compounds.

## 2 Materials and Methods

### 2.1 Chemicals and Instrumentation

The instrumental and equipment resources are as follows: a Varian CARY 100 UV–vis spectrophotometer (Mulgrave, Victoria, Australia) equipped with matched HELLMA quartz cuvettes with a 10 mm light path, a Radwag AS 220/C/2 analytical balance (Radom, Poland), a Sonorex ultrasonic bath (Bandelin, Germany), a Velp Scientifica ZX3 Advanced Vortex Mixer (Usmate, Italy), a Memmert water bath WNB 7-45 (Schwabach, Germany), and a HANNA HI 221 pH meter (Woonsocket, RI-USA).

Chemicals used in this study were of analytical grade purity and were purchased from different sources. Cobalt(II) sulfate heptahydrate, sodium sulfite, disodium hydrogen phosphate dihydrate, sodium dihydrogen phosphate dihydrate, and ammonium chloride were supplied by Merck; acetic acid, sodium acetate, ammonia, ethanol (EtOH), methanol (MeOH), acetonitrile, acetone, *tert*-butanol, 3,3′,5,5′-tetramethylbenzidine (TMB), gallic acid (GA), ferulic acid (FA), catechin (CAT), epicatechin (EC), ascorbic acid (AA), l-cysteine (CYS), caffeic acid (CFA), glutathione (GSH), NAC, quercetin (QR), *p*-coumaric acid (*p*-CUM), and naringenin (NG) were from Sigma-Aldrich; and rhodamine B was from Sigma.

### 2.2 Preparation of Solutions

#### 2.2.1 Solutions Used in the Determination of SORs

Cobalt(II) sulfate heptahydrate (0.2 M) and sodium sulfite (2.0 × 10^–3^ M) were prepared by dissolving appropriate amounts of solids in distilled water. The TMB solution at 3.0 × 10^–3^ M concentration was prepared in EtOH. The NH_3_/NH_4_Cl pH 8 buffer solution was prepared by mixing appropriate amounts of aliquots taken from 1.0 M NH_3_ and NH_4_Cl solutions. In order to prepare the pH 4 buffer solution, appropriate volumes were taken from acetic acid and sodium acetate (both 0.5 M) and mixed. For rhodamine B, the stock solution at a concentration of 1.0 × 10^–3^ M was prepared by dissolving an appropriate amount of solid in distilled water, and then, a working solution of 2.5 × 10^–5^ M concentration was prepared by dilution with distilled water.

#### 2.2.2 Antioxidant (AOx) Solutions

Stock solutions were prepared as follows: 0.01 M GA, FA, QR, CFA, *p*-CUM, NG, CAT, and EC were prepared by dissolving appropriate amounts of solid substance in ethanol. GSH, AA, and NAC (all at 0.01 M) were prepared in distilled water. To prepare 0.01 M CYS, the weighed solid material was dissolved in 0.5 mL of 1.0 M HCl and diluted to 25 mL with distilled water. The stock solutions of AOx prepared in ethanol were stored at −18 °C, and working solutions were prepared by dilution. Thiol-type AOx (GSH, NAC, and CYS) and AA were freshly prepared before the experiments.

#### 2.2.3 Preparation of Real Samples

White tea, green tea, and black tea (i.e., minimally processed, steamed/dried but unfermented, and fully fermented leaves of *Camellia sinensis*, respectively) were used as real examples. In order to prepare related sample solutions, ready-to-use tea bags purchased from local markets were used. Samples were weighed and then prepared as described earlier by Apak et al.^14^

### 2.3 Proposed Method for the Generation and Determination of SORs

All combinations of Co(II) and SO_3_^2–^ (i.e., individually and together) were tested to optimize the generation of SORs to ensure that TMB coloration arose from SOR.

#### 2.3.1 Generation of SORs in the Presence or Absence of Scavengers

0.5 mL of 0.2 M CoSO_4_·7H_2_O + 0.5 mL of pH 8 NH_3_/NH_4_Cl buffer solution + 2.0 mL of distilled water + (100 – *x*) μL of EtOH (for antioxidant solutions prepared in water (2 – *x*) mL of water + 100 μL of EtOH) + *x* μL of AOx + 0.4 mL of 2.0 × 10^–3^ M sodium sulfite solution were mixed in this order, the mixture was incubated at 37 °C for 30 min, and 0.3 mL of EtOH was added to the resulting mixture to stop the reaction.

#### 2.3.2 Determination of Resulting SORs in the Final Solution

1.0 mL of the incubation solution was taken, to which 1.0 mL of pH 4 acetic acid/sodium acetate buffer solution and 0.1 mL of 3.0 × 10^–3^ M TMB solution were added in this order and incubated at 50 °C for 10 min in a water bath. The solution was allowed to reach room temperature for about 10 min. Finally, the absorbance of the resulting solution was measured at 653 nm against a reagent blank.

### 2.4 Optimization of SOR Generation for the Proposed TMB Method

To determine the optimal parameters for radical generation, appropriate amounts of Na_2_SO_3_, CoSO_4_, TMB reagent, pH, incubation temperature, and time were tested by changing one parameter at a time while keeping the others constant.

#### 2.4.1 Optimization of Na_2_SO_3_ and CoSO_4_ Concentrations

In order to optimize Na_2_SO_3_ concentration, different volumes ranging from 0.1 to 0.5 mL were taken from the sulfite solution at a concentration of 2.0 mM. Similarly, volumes ranging from 0.1 to 0.9 mL were taken from 0.2 mM CoSO_4_ solution for optimization of CoSO_4_ concentration. The rest of the method was applied, as described in Section 2.3.

#### 2.4.2 Optimization of TMB Concentration

To determine the best TMB concentration, the 0.01 M stock solution was diluted at different ratios ranging from 3- to 60-fold. Then, a volume of 0.1 mL was taken from the diluted solutions and added to a reaction mixture consisting of 1.0 mL of incubated solution and 1.0 mL of pH 4 buffer solution. The resulting mixture was incubated in a water bath at 50 °C for 10 min and allowed to reach room temperature, and its absorbance was measured at 653 nm.

#### 2.4.3 Optimization of pH

In order to determine the optimal pH for generation of SORs, a series of buffer solutions at different pH values were prepared as follows: pH 3 buffer solution by using citric acid and sodium citrate; pH 4.0–6.0 buffers by mixing acetic acid and sodium acetate; and pH 8–9 buffer solutions by mixing aq. NH_3_ and NH_4_Cl solutions. For pH 7 buffer, NaH_2_PO_4_ and Na_2_HPO_4_ were used (all buffer solutions consisting of acid and conjugate base were at 1.0 M concentration, except pH 7.0 buffer, which was at 0.5 M). The method described in Section 2.3 was applied by using 0.5 mL of the chosen buffer (pH varying between 3 and 10) instead of the mentioned pH 8 NH_3_/NH_4_Cl buffer. The same experimental procedure was adapted to the optimization of pH for SOR determination by replacing 1.0 mL of pH 4 buffer (Section 2.3) with 1.0 mL of buffer at the chosen pH.

#### 2.4.4 Determination of Optimal Reaction Time

For the generation of SOR, the reaction mixture was incubated in a water bath at 37 °C for different time intervals between 5 and 60 min. For measuring the generated SOR, radical generation was repeated as described in Section 2.3; however, after the addition of the TMB reagent, the reaction mixture was kept in a water bath at 50 °C for 5, 15, 30, and 60 min.

#### 2.4.5 Determination of Optimal Incubation Temperature

The incubation periods for radical production and determination were different, as explained in Section 2.3. Accordingly, temperature optimization experiments were performed for each of these two steps. To determine the optimal temperature for radical generation, different temperatures between 25 and 50 °C were tested, whereas for radical determination, the temperatures were varied between 25 and 75 °C.

### 2.5 Investigation of the Effects of Solvents and Other ROS

#### 2.5.1 Effects of Solvents

In order to observe the effects of different solvents on the proposed TMB method, the method detailed in Section 2.3 was repeated in the presence and absence of 0.1 mL of EtOH, MeOH, acetone, acetonitrile, and *tert*-butanol separately (the tested solvents were not diluted); the solvent effect was evaluated by absorbance measurements.

#### 2.5.2 Effects of Other ROS

In order to investigate possible interferences that may arise from different ROS, three colorimetric tests were conducted. The presence of hydroxyl radicals was tested with a modified CUPRAC colorimetric reaction using a salicylate probe for obtaining dihydroxybenzoic acids responding to the cupric–neocuproine reagent.^15^ Nitro blue tetrazolium chloride (NBT) test was used to investigate the presence of superoxide anion radicals^16^ and 1,3-diphenylisobenzofuran (DPBF) test for singlet oxygen,^17^ as briefly described below.

NBT test for superoxide anion radicals: in order to test O_2_^•–^, the method described for SORs was repeated in the presence of NBT. Accordingly, 0.5 mL of 0.2 M CoSO_4_·7H_2_O + 0.5 mL of pH 8 NH_3_/NH_4_Cl buffer solution + 1.0 mL of distilled water + 100 μL of ethanol (EtOH) + 1.0 mL of NBT + 0.4 mL of 2.0 × 10^–3^ M sodium sulfite solution were mixed in this order; the mixture was incubated at 37 °C for 30 min, and 0.3 mL of EtOH was added to the resulting mixture to stop the reaction. Superoxide anion radicals, if present, are expected to yield a characteristic blue-purple color measurable at 560 nm.

DPBF test for singlet oxygen: 0.5 mL of 0.2 M CoSO_4_·7H_2_O + 0.5 mL of pH 8 NH_3_/NH_4_Cl buffer solution + 2.0 mL of distilled water + 100 μL of EtOH + 0.4 mL of 2.0 × 10^–3^ M sodium sulfite solution were mixed in this order; the mixture was incubated at 37 °C for 30 min, and 0.3 mL of EtOH was added to the resulting mixture to stop the reaction. On the other hand, the same operation was repeated in the absence of Co(II) and Na_2_SO_3_ in order to hinder radical generation. This reagent mixture was used as a reference blank.

At the end of the incubation period, an aliquot of 1.0 mL was taken from each solution, and 1.0 mL of EtOH and 50 μL of the DPBF reagent (dissolved in EtOH) were added. The absorbance of the final reaction mixture was measured at 410 nm. Here, singlet oxygen—if generated—is expected to cause a decrease in the color intensity of the DPBF reagent.

### 2.6 Determination of SOR Scavenging Activities of Selected AOx Compounds Using the Proposed TMB Method

The SOR scavenging effects of AOx compounds belonging to different subclasses were investigated by testing the scavenging activities of 12 different AOx compounds, namely, QR (flavonoid), NG (flavanone), GA (hydroxybenzoic acid), *p*-CUM, CFA, FA (hydroxycinnamic acid), CAT, EC (flavanol), GSH, CYS, NAC (thiol), and AA. For this purpose, working solutions of AOx compounds were set in the concentration range of 0.2–5.0 mM, and volumes ranging from 20 to 100 μL were taken. Then, the method in Section 2.3 was applied in the absence and presence of AOx compounds at different concentrations.

For investigating the possible additive effects of AOx compounds in SOR scavenging, a number of binary and ternary AOx mixtures were prepared and tested using the proposed TMB method. For this purpose, 20 μL of 1.0 mM GA, 10 μL of 0.5 mM CFA, 10 μL of 5.0 mM CYS, 20 μL of 0.1 mM CAT, 200 μL of 0.2 mM AA, and 20 μL of 0.5 mM FA were used to prepare binary and ternary mixtures at different combinations.

### 2.7 Rhodamine B Decolorization Assay for SOR Determination

To produce SORs, the first part of the procedure explained in Section 2.3 was likewise applied with one exception: the sulfite solution was 20 mM instead of 2.0 mM. Then, 1.0 mL of 1.25 × 10^–5^ M RhB solution + 1.0 mL of pH 4 acetic acid/sodium acetate buffer solution were mixed in this order, and 1.0 mL of incubation solution was added into this mixture. The final mixture solution was incubated in a water bath at 50 °C for 10 min and allowed to reach room temperature, and the resulting absorbance was measured at 554 nm.

#### 2.7.1 Optimization of the Rhodamine B Decolorization Assay

For this purpose, the most appropriate amount of RhB was determined. The color intensity of RhB can change depending on the freshness of the RhB reagent. In our case, a RhB solution at a concentration of 1.25 × 10^–5^ M was used, whose tested volume ranged within 0.1–1.2 mL.

The second parameter to be set was the sulfite concentration. The Na_2_SO_3_ amount used in the TMB method was not sufficient for an effective decolorization. Therefore, the Na_2_SO_3_ concentration was increased to 20 mM, and different volumes changing between 0.1 and 0.8 mL were withdrawn from this solution, followed by the application of the RhB method described in Section 2.7.

#### 2.7.2 Determination of SOR Scavenging Activities of Selected AOx Compounds Using the Reference RhB Assay

The same AOx compounds mentioned in the TMB method were tested with the RhB assay to compare the results. The solutions of the tested compounds were diluted to different concentrations between 0.2 and 10 mM, and different volumes between 20 and 100 μL were taken for analysis (as described in Section 2.7) in the presence and absence of AOx compounds.

### 2.8 Determination of SOR Scavenging Activities of Real Samples

White, green, and black tea samples were used as the real samples, of which the infusions were diluted at different ratios just before analysis. The white tea sample was diluted 20 times for both the TMB and RhB methods. On the other hand, black and green tea samples were diluted 10 times for the TMB method and 5 times for the RhB method. The volume taken was 0.3 mL for all samples. Experiments were repeated five times to report the findings after statistical evaluation.

## 3 Results and Discussion

**Figure 1:**
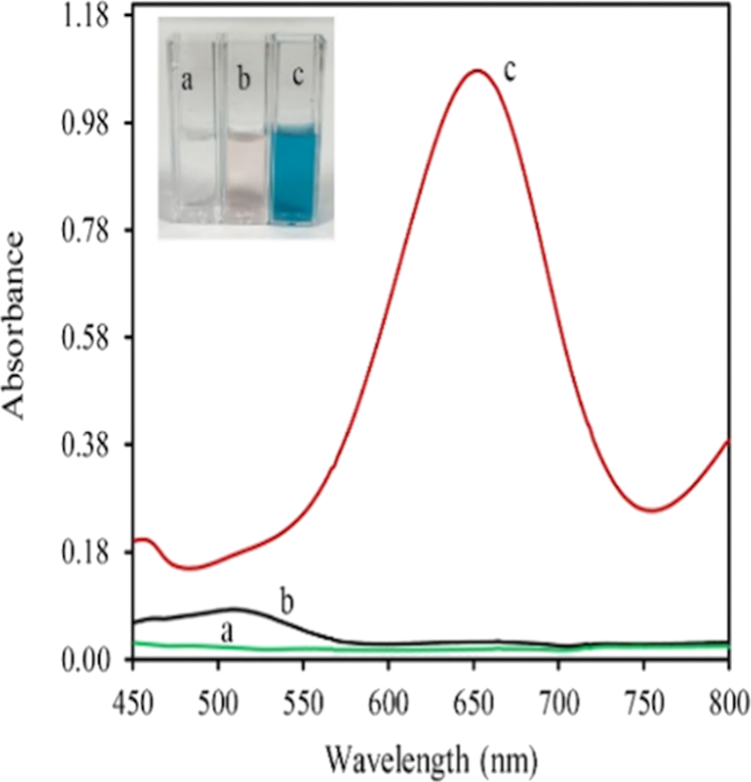
Visible spectra of TMB after treatment with (a) sulfite (without Co(II)); (b) Co(II) (without sulfite); and (c) both Co(II) and sulfite (inset: solution colors).

To ensure that the TMB oxidation was caused by SORs and not sulfite, the method was applied in three different ways, namely, in the presence of (i) only Co(II) (without SO_3_^2–^), (ii) only SO_3_^2–^ (without Co(II)), and (iii) Co(II) in conjunction with SO_3_^2–^. The visible spectra for the three cases are listed in Figure 1.

As can be seen from Figure 1, the intense blue color originating from TMB oxidation (i.e., due to the charge-transfer complex composed of TMB and its two-electron oxidation product, diimine)^18^ could only be produced from SOR attack on TMB, requiring the participation of both Co(II) and sulfite. The pale pink color seen in the inset image of Figure 1 (spectral cuvette (b)) originated from Co(II) in solution as a result of d–d transitions. This showed that TMB oxidation resulted from SOR attack and not from sulfite alone.

### 3.1 Determination of Optimal Experimental Parameters for the TMB Method

#### 3.1.1 Optimization of Na_2_SO_3_ and CoSO_4_ Concentrations for SOR Generation

**Figure 2:**
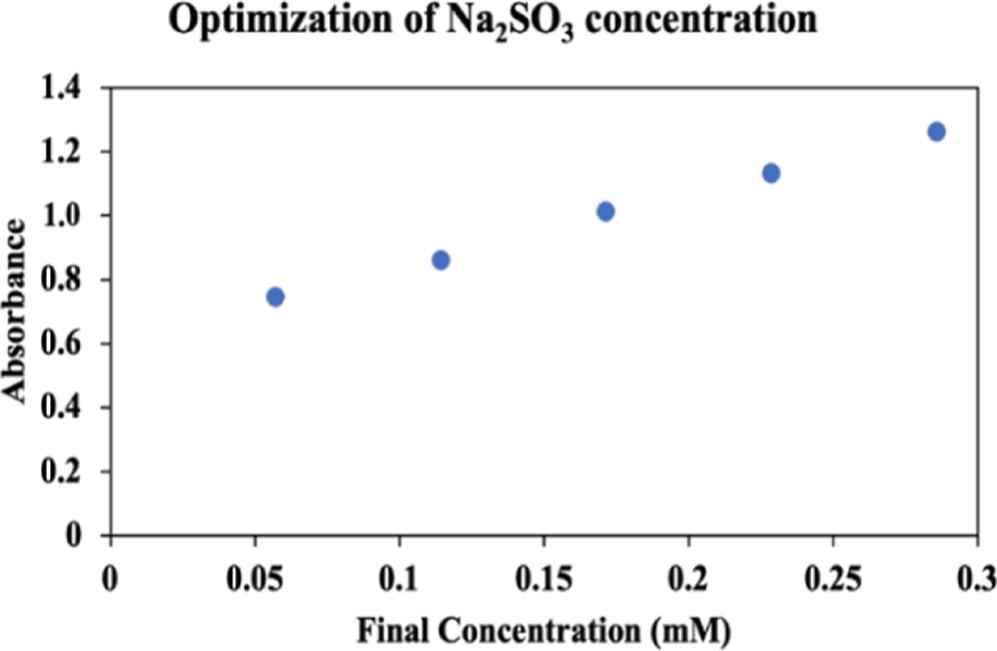
TMB absorbances recorded against final concentrations of Na_2_SO_3_ in the reagent mixture used for SOR generation.

The experiments were conducted as described in Section 2.4. According to the obtained data, the absorbance values measured against the final concentrations of Na_2_SO_3_ are given in Figure 2.

Data in Figure 2 shows that the absorbance increase continued with increasing concentrations of Na_2_SO_3_. However, chemical deviations from Beer’s law of optical density are more common at absorbance values higher than 1.0 due to intermolecular interactions in concentrated solutions. Accordingly, a volume of 0.4 mL taken from the 2.0 mM Na_2_SO_3_ solution was determined as the best value for sulfite.

**Figure 3:**
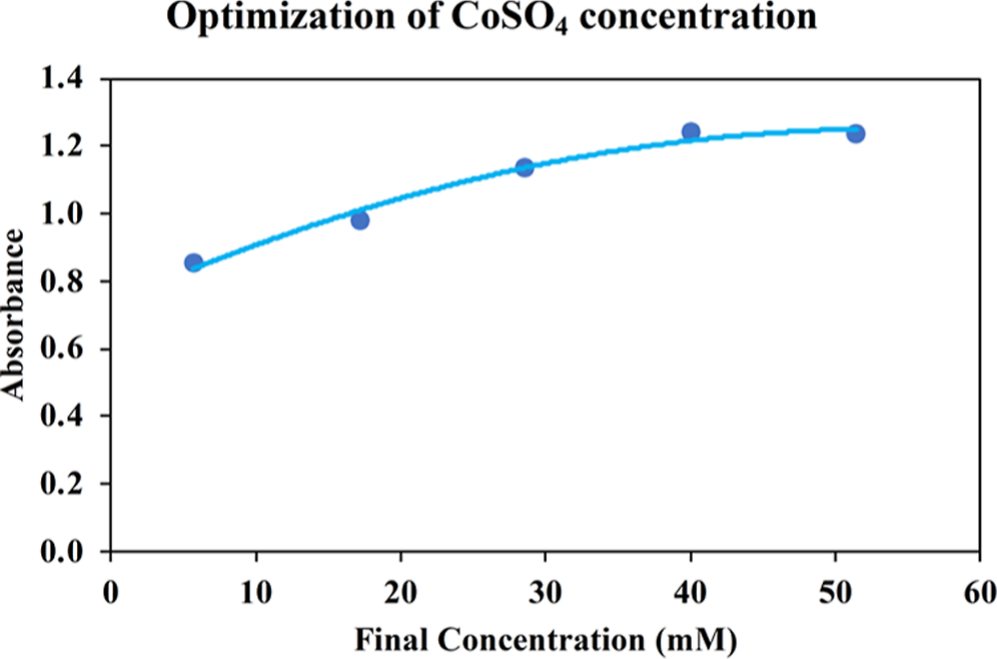
TMB absorbances recorded against final concentrations of CoSO_4_ in the reagent mixture used for SOR generation.

The TMB absorbances recorded by the standard procedure were plotted against CoSO_4_ concentrations for SOR generation, as shown in Figure 3.

According to the plot in Figure 3, when the final concentration of CoSO_4_ was between 5.5 and 40 mM, the absorbance values increased and then remained almost the same. Consequently, 0.5 mL of 0.2 M CoSO_4_ was determined as the best value for further experiments.

#### 3.1.2 Optimization of TMB Concentration

**Figure 4:**
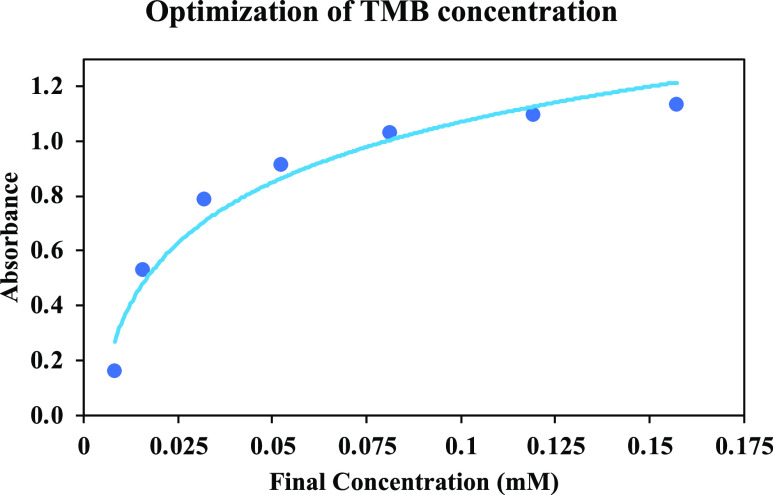
Absorbances recorded against the final concentrations of TMB in the reagent mixture used for the determination of SORs.

The dependence of the absorbance on the final concentration of TMB (using the procedure given in Section 2.4) is given in Figure 4.

As can be seen from Figure 4, the relationship between the final concentrations of TMB and absorbance values was not linear (unlike those observed in Figures 2 and 3). Therefore, the concentration yielding maximal absorbance was chosen for further experiments; that is, a volume of 0.1 mL was taken from TMB solution at a concentration of 3.0 × 10^–3^ M

#### 3.1.3 Optimization of pH

The optimal pH values were set for both parts of the method, namely, the generation and determination of SORs. As stated in Section 2.4, pH values between 3 and 9 were tested separately for radical generation and determination. In the investigation of the optimal pH value for radical generation, the highest absorbance value was obtained at pH 8 in the presence of NH_3_/NH_4_Cl buffer. In the second step regarding the determination of the generated SORs, it was observed that acidic conditions gave better results, and pH 4 obtained by acetic acid/sodium acetate buffer was chosen as the optimal pH.

Although the radical generation mechanism during metal-catalyzed S(IV) autoxidation is very complex, there is some evidence that it starts *via* a metal–sulfito complex formation. Then, there are different reactions involving the decomposition of this complex due to the formation of radical species (such as SO_5_^•–^) in the presence of oxygen. During these reactions, pH is an important parameter since the distribution of metal ions and sulfur(IV) species are pH dependent that may show different reactivities. It is known that SO_3_^2–^ is more reactive than HSO_3_^–^.^19^ In accordance with this, the optimal pH value, set to pH 8, was higher than the p*K*_a2_ value (7.21) for H_2_SO_3_, where the predominant S(IV) species was SO_3_^2–^.

When it comes to the determination of SOR using TMB, it is known that this reagent is commonly applied in a slightly acidic medium.^20^ Our results showed that pH 4 was the optimal value for determining SORs using the TMB reagent.

#### 3.1.4 Optimization of Reaction Time

The proposed procedure was applied, as stated in Section 2.4. The obtained results showed that a time period of 30 min for radical generation and 10 min for measurement (following the addition of TMB) were optimal (data not shown). At the second step of the experiments (in the determination of the generated radical), an absorbance value slightly above 1.0 was measured in accordance with the recommended procedure in Section 2.3.

#### 3.1.5 Optimization of Incubation Temperature

**Figure 5:**
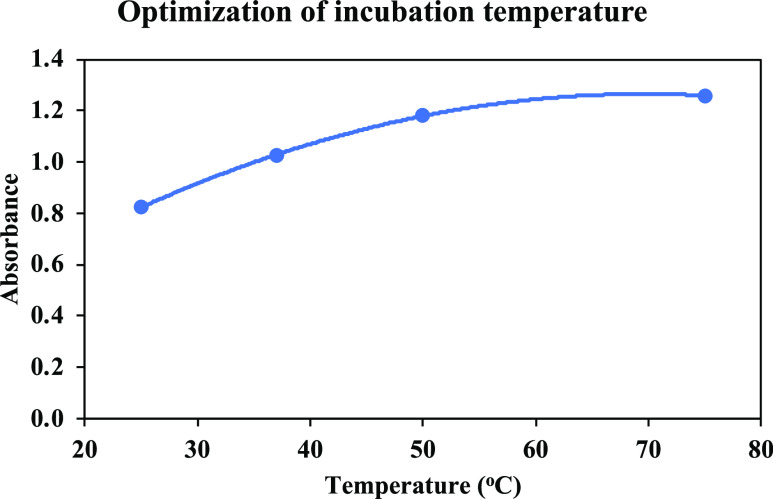
Absorbance measured against the incubation temperature after TMB addition.

The procedure described in Section 2.4 was applied, and the results showed that a temperature of 37 °C was sufficient for producing SORs at an appreciable rate. On the other hand, the color intensity increased with increasing temperature after TMB addition. The obtained results are given in Figure 5.

As can be seen from Figure 5, the absorbance increased steadily between 25 and 50 °C and then slowly up to 70 °C. Therefore, 50 °C was chosen as the optimal temperature and applied for further experiments.

#### 3.1.6 Evaluation of Solvent Effects on the Proposed TMB Method

Examination of solvent effects enables one (i) to choose the best solvent for AOx determination and (ii) to decide whether the generation/scavenging of correct radicals (in this case, SORs) is made to achieve method selectivity (e.g., MeOH and EtOH are alcohols to effectively scavenge hydroxyl radicals). The obtained results are given in Table 1.

**Table 1:** Effects of Different Solvents on the Proposed TMB Method

solvent tested	*A*_TMB_
no extra solvent	1.0492
ethanol	0.9341
methanol	0.8915
acetone	0.1120
acetonitrile	1.0560
*tert*-butanol	1.0372

#### 3.1.7 Investigation of Possible Interferences of Different ROS

As briefly described in Section 2.5.2, selective tests for the determination of hydroxyl radicals *via* hydroxylation of the salicylate probe to produce dihydroxybenzoates detectable by CUPRAC colorimetry and HPLC, for the estimation of superoxide anion radicals with the NBT reagent, and for the detection of singlet oxygen with the DPBF reagent gave negative results, confirming that only SORs were generated or remained stable in the test solution under optimized conditions, as formulated by Coddens *et al*.^21^

#### 3.1.8 Complexation/Chelation Effects

To see the effect of cobalt chelation, EDTA (as disodium salt) was added in an equivalent amount to Co(II), and the conventional procedure was followed to see if SORs were generated under identical conditions. However, Co(II) having d^7^ electron configuration is easily oxidized to Co(III) by virtue of its coordination properties in the presence of EDTA as a high ligand-field chelator (the six d-electrons of Co(II) would be paired in lower t_2g_ electronic orbitals, and there would remain only one unpaired electron in the higher energy e_g_ level, which would be easily lost by the Jahn–Teller effect distorting the octahedral geometry. Therefore, it is extremely difficult to keep Co(II) in the divalent state in the presence of a strong-field ligand such as EDTA). This would mean that the established procedure for generating SORs with the reaction between sulfite and Co(II) would not work. This was confirmed experimentally to see that the TMB solution was not colored, confirming that SORs were not generated with Co-EDTA and sulfite.

Data depicted in Table 1 revealed that among the solvents tested, only acetone caused an important decrease in the absorbance of TMB. This decrease can be interpreted as inhibition of the generation of SORs in the presence of acetone or the SOR scavenging effect of acetone. Acetone is an effective solvent in free radical scavenging, as it was reported that ABTS cationic radicals (ABTS^•+^) were transformed to their reduced form (ABTS) by acetone.^22^ Choi *et al*. reported acetone decomposition from electronic industry wastewater using ^•^OH obtained from the reaction between Fe- and Al-immobilized catalysts and H_2_O_2_.^23^ A reaction between the formed radicals and acetone may decrease the total radical content and cause a serious reduction in the observed color intensity of TMB in the presence of oxidative radical species. In another example study by Banat et al., it was reported that acetone proved to be a good photosensitized material capable of completely decolorizing methylene blue-containing wastewater.^24^

Among the tested solvents, *tert*-butanol and ethanol were utilized for differentiation of hydroxyl and sulfate radicals in a previous study of the authors.^12^ Anipsitakis and Dionysiou reported a similar result in that while ethanol could quench sulfate radicals at a high ratio even in the diluted form, *tert*-butanol was much less effective on sulfate radicals. On the other hand, both alcohol solvents were effective on hydroxyl radical scavenging.^10^ This phenomenon can be explained by the different radical scavenging effects of α-hydrogen-bearing alcohols such as ethanol and alcohols lacking an α-hydrogen such as *tert*-butanol.^25^ Our findings confirmed that there was no appreciable ^•^OH formation in the proposed Co(II)/Na_2_SO_3_ system. We also tested the presence of hydroxyl radicals with a salicylate probe to see whether it was hydroxylated to dihydroxybenzoates that should positively respond to the CUPRAC reagent, but the test gave a negative result with the cupric–neocuproine complex, proving the absence of ^•^OH under our experimental conditions.^15^ On the other hand, the radicals generated in our system are not composed of a single species but comprise a mixture of different SORs. The slight absorbance decrease observed with ethanol and methanol (Table 1) may result from the scavenging of a small part of the generated radicals.

### 3.2 Optimization of the RhB Decolorization Assay

**Figure 6:**
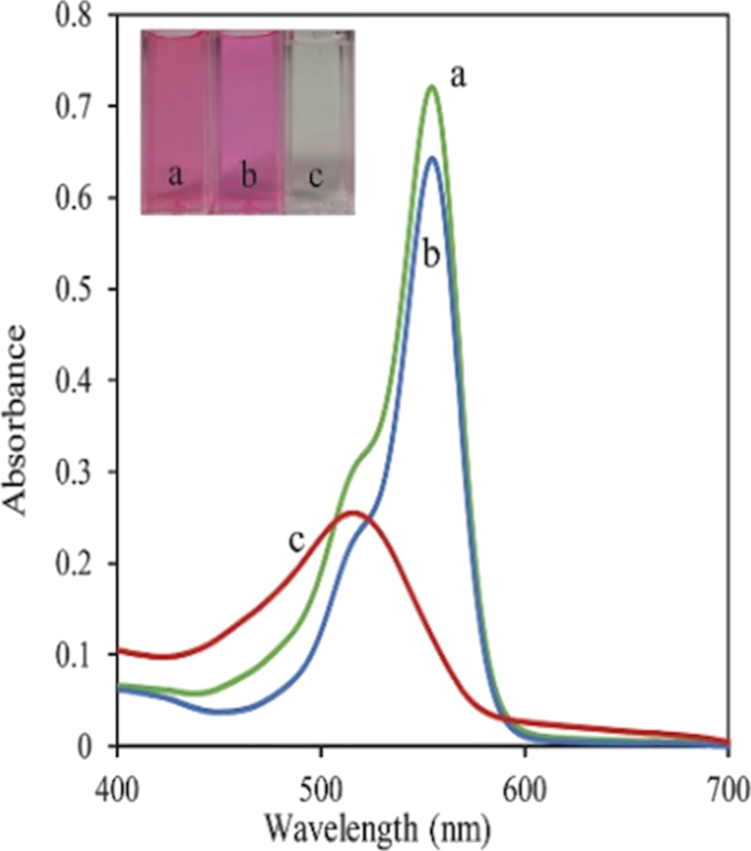
Absorption spectra of 3.3 × 10^–6^ M RhB recorded in the presence of (a) only Co(II), (b) only sulfite, and (c) Co(II) + Na_2_SO_3_ (inset: solution colors).

The RhB decolorization assay was applied in the presence of (i) only Co(II), (ii) only Na_2_SO_3_, and (iii) Co(II) + Na_2_SO_3_. The results are listed in Figure 6.

As can be seen from Figure 6, neither Co(II) nor sulfite could decolorize RhB solution, but their combination was able to do so due to the generation of SORs. RhB is one of the most resistant organic compounds against oxidative decomposition, and it can be decomposed only by means of highly powerful oxidants such as hydroxyl and sulfate radicals (both having standard redox potentials exceeding 2 V). Therefore, the RhB decolorization assay can be accepted as an appropriate method for comparison.

In the RhB method, the optimal RhB amount was determined as 1.0 mL taken from 1.25 × 10^–5^ M solution, which yielded an absorbance of about 0.7. In order to determine the optimal concentration of Na_2_SO_3_ solution for SOR generation, the procedure summarized in Section 2.7 was followed. It was observed that for Na_2_SO_3_ volumes between 0.1 and 0.4 mL, the absorbance of RhB increased sharply, while for higher volumes of Na_2_SO_3_, the RhB absorbance only slightly increased. So, 0.4 mL was chosen as the optimal value. Since the decrease in the color intensity of RhB is due to the presence of strong oxidants in the reaction medium, this can be interpreted as an indicator of radical formation.

### 3.3 Determination of SOR Scavenging Activities of Selected Antioxidant Compounds

Sulfur dioxide is known to be easily taken up by humans from atmospheric sources and sulfited foods, and it can combine with Co(II) ions released from the degradation of vitamin B12 or orthopedic implants to cause toxic effects through SOR generation. The combination of Co(II) with sulfite in wastewater disinfection was shown to rapidly inactivate viable bacteria regardless of bacterial species and cell density.^26^ Thus, the role of AOx to relieve Co(II)/sulfite toxicity through scavenging SORs needs to be explored. For this purpose, a series of AOx compounds were investigated in terms of SOR scavengers.

#### 3.3.1 Investigation of Radical Scavenging Activities of AOx Compounds Using the Proposed TMB Method

The data in Figure 1, already containing the visible spectra of TMB after treatment with only Na_2_SO_3_, only Co(II), and Co(II) + Na_2_SO_3_ combination, were enriched with the spectra of antioxidant (AOx)-containing combinations, namely, only AOx, Co(II) + AOx, Na_2_SO_3_ + AOx, and Co(II) + Na_2_SO_3_ + AOx; the AOx used in the experiments was GA with a final concentration of 8.2 μM. These additional spectra (not shown) demonstrated that only two of the mixtures (Co(II) + Na_2_SO_3_ and Co(II) + Na_2_SO_3_ + AOx) could produce oxidized TMB (or its colored charge-transfer complex) to differing extents. The TMB peak resulting from the Co(II) + Na_2_SO_3_ combination was decreased in the ternary mixture containing AOx, that is, Co(II) + Na_2_SO_3_ + AOx. It was concluded that only the radicals generated from {Co(II) + Na_2_SO_3_} were reactive against TMB, and as expected, the presence of antioxidants mitigated the formation of this charge-transfer complex as a result of their radical scavenging action.

To determine the effect of phenolic complexation of Co(II) on the proposed method, two sets of experiments were conducted. So, (i) Co(II), GA, and Na_2_SO_3_ were added at the beginning, and these were incubated together; (ii) Co(II) and Na_2_SO_3_ were incubated first to allow time for radical formation, and then scavenger AOx was added to it. Both tests gave almost identical calibration equations (within experimental error limits) as Δ*Aversus* AOx concentration to conclude that AOx compounds essentially scavenged the generated radicals due to the simple reason that scavenging of radicals by AOx is much faster than complexation of radical generators (in this case, Co(II), in the presence of sulfite) by antioxidants.

In AOx testing against free radicals, there is always an ambiguity whether AOx compounds actually scavenge free radicals or merely complex the metal ions in the test system so as to hinder radical formation. Halliwell states that if the AOx is simply acting by chelation, it will not be consumed during the reaction, shown by HPLC (or another objective) technique; in other words, the AOx should be chemically modified if it reacts with the reactive species of concern.^27^ GA was tested as an AOx in the optimized system, as described in Section 2.6. The structural change of GA was followed with RP-HPLC using a C18 column and gradient elution with a mobile phase consisting of methanol and H_3_PO_4_ solution at 0.2%. In the chromatogram (not shown), the GA peak at 3.62 min retention time was significantly reduced, showing GA consumption after the reaction with SORs. By the definition proposed by Halliwell, this is a proof that the AOx acted as a radical scavenger and not merely as a chelating agent for cobalt (that would hinder radical generation).^27^

Typically, two different-colored products are formed in the course of oxidation of TMB; the first colored product is a blue charge-transfer complex of the parent diamine and the diimine oxidation product, which exists in rapid equilibrium with the radical cation.^28^ TMB reacts with an oxidizing agent to yield a radical cation (TMB^•+^) which forms a blue charge-transfer complex (having an abs. max. at 653 nm) with a second TMB molecule.^29^ Although further oxidation is possible, TMB^•+^ is relatively stable unless it is reacted with strong reductants over prolonged exposure. The mechanism of oxidation and subsequent colored product formation from TMB has been well documented and only occurs through the formation of reactive species. In this work, these reactive species are SORs. Thus, a decrement in the color intensity of the blue charge-transfer complex of TMB is due to the quenching of SORs by AOxs thereby causing less charge-transfer complex to form, rather than the chemical reduction of oxidized TMB by antioxidant compounds (which would be kinetically much slower than free radical scavenging by antioxidants). The reactions involved in antioxidant–sulfate radical and TMB–sulfate radical couples are very fast; TMB, once oxidized to the TMB^•+^ radical cation, can undergo a comparably much slower chemical reduction with sulfite anions^30^ and other reducing agents (e.g., phenolic antioxidants). That is why, the Co(II)–sulfite couple may be used in the generation of SORs which rapidly oxidize TMB to the (TMB^•+^–TMB) charge-transfer complex, but the reaction is not reversible by chemical reduction back to colorless TMB with the reductant sulfite excess existing in the medium. The same is true for phenolic antioxidants.

In this method, since TMB was oxidized to a blue charge-transfer complex (abbreviated as ox-TMB) by means of the generated radicals, the presence of AOx caused a decrease in the measured absorbance value of ox-TMB *via* the SOR scavenging action of AOx compounds. The decrement in color intensity was directly proportional to the final concentration of AOx. The calibration graphs between absorbance decrease (Δ*A*) and final concentrations of tested AOx were drawn. Δ*A* was calculated by the equation
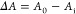
where *A*_0_ is the absorbance measured in the absence of AOx and *A*_i_ is the absorbance measured in the presence of AOx. For all of the tested AOx compounds, linear working ranges, LOD, LOQ values, and the equation of the calibration graphs were collected and are presented in Table 2.

**Table 2:** Linear Working Ranges, LOD and LOQ Values, and Calibration Equations with Correlation Coefficients of AOx Compounds Tested by the Proposed TMB Method

AOx	equation of calibration graph	linear ranges (M)	LOD (M)	LOQ (M)	determination coefficient
GA	Δ*A* = 6.53 × 10^4^ C + 0.154	2.72 × 10^–6^ to 1.36 × 10^–5^	3.59 × 10^–7^	1.20 × 10^–6^	*R*^2^ = 0.9962
CAT	Δ*A* = 1.99 × 10^5^ C + 0.088	5.44 × 10^–7^ to 1.90 × 10^–6^	1.17 × 10^–7^	3.91 × 10^–7^	*R*^2^ = 0.9965
AA	Δ*A* = 1.70 × 10^4^ C + 0.197	5.44 × 10^–6^ to 1.90 × 10^–5^	1.38 × 10^–6^	4.60 × 10^–6^	*R*^2^ = 0.9758
EC	Δ*A* = 6.14 × 10^5^ C + 0.164	1.36 × 10^–7^ to 1.36 × 10^–6^	3.82 × 10^–8^	1.27 × 10^–7^	*R*^2^ = 0.9996
FA	Δ*A* = 6.19 × 10^4^ C + 0.064	1.36 × 10^–6^ to 6.80 × 10^–6^	3.78 × 10^–7^	1.26 × 10^–6^	*R*^2^ = 0.9922
CYS	Δ*A* = 1.69 × 10^4^ C + 0.073	6.80 × 10^–6^ to 3.40 × 10^–5^	1.39 × 10^–6^	4.63 × 10^–6^	*R*^2^ = 0.9979
CFA	Δ*A* = 1.59 × 10^5^ C – 0.043	6.80 × 10^–7^ to 4.76 × 10^–6^	1.47 × 10^–7^	4.91 × 10^–7^	*R*^2^ = 0.9718
GSH	Δ*A* = 1.51 × 10^4^ C + 0.096	6.80 × 10^–6^ to 3.40 × 10^–5^	1.38 × 10^–6^	4.61 × 10^–6^	*R*^2^ = 0.9923
NAC	Δ*A* = 1.69 × 10^4^ C + 0.107	1.36 × 10^–5^ to 4.90 × 10^–5^	3.37 × 10^–6^	1.13 × 10^–5^	*R*^2^ = 0.9851
QR	Δ*A* = 8.88 × 10^4^ C – 0.018	1.36 × 10^–6^ to 4.76 × 10^–6^	2.64 × 10^–7^	8.80 × 10^–7^	*R*^2^ = 0.9689
NG	Δ*A* = 1.22 × 10^4^ C – 0.039	6.80 × 10^–6^ to 6.80 × 10^–5^	1.92 × 10^–6^	6.39 × 10^–6^	*R*^2^ = 0.9841
p-CUM	Δ*A* = 7.47 × 10^3^ C + 0.094	1.36 × 10^–5^ to 6.80 × 10^–5^	3.14 × 10^–6^	1.05 × 10^–5^	*R*^2^ = 0.9602

As can be seen from Table 2, the LOD values for tested AOx compounds were between 0.04 and 3.37 μM. These very low values show that the proposed method can be used successfully in the determination of the SOR scavenging capacity of the AOx compounds.

#### 3.3.2 Investigation of SOR Scavenging Effectiveness of AOx Compounds Using the RhB Decolorization Method

It should be noted that the mechanism of the reference method is different from that of the TMB method. Here, the intact RhB solution was bright pink, and the generated radicals caused a decrease in color. Hence, the presence of AOx compounds caused an increase in the measured absorbance value, and it was determined that this absorbance difference was proportional to the AOx concentration. Here, Δ*A* was calculated by the equation
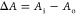
where *A*_RhB_ is the absorbance measured with pure RhB (without SOR and AOx), *A*_i_ is the absorbance measured in the presence of RhB + SOR + AOx, and *A*_0_ is the absorbance measured in the presence of RhB + SOR (without AOx).

The absorbance measured with pure RhB (*A*_RhB_) was evaluated to determine the relative efficiency of the AOx action. The approach in the calculation can be summarized as follows: *A*_RhB_—*A*_0_ corresponds to the maximum absorbance difference between the absorbance of intact RhB and of that under SOR attack (without any AOx in the reaction medium). On the other hand, the presence of AOx at different concentrations caused an increase in absorbance (*A*_i_) compared to *A*_0_. Thus, the relative increment of absorbance in the presence of the AOx was Δ*A* = *A*_i_ – *A*_o_, reflecting the protective effect of AOx on radical-induced RhB decolorization. The equations found for calibration graphs, linear working concentration ranges, and LOD and LOQ values were collected and are shown in Table 3.

**Table 3:** Linear Working Ranges, LOD and LOQ Values, and Calibration Equations with Correlation Coefficients of AOx Compounds Tested by the Reference RhB Method

AOx	equation of calibration graph	linear ranges (M)	LOD (M)	LOQ (M)	determination coefficient
GA	Δ*A* = 0.88 × 10^4^ C – 0.0471	9.52 × 10^–6^ to 4.76 × 10^–5^	2.76 × 10^–6^	9.2 × 10^–6^	*R*^2^ = 0.9917
CAT	Δ*A* = 0.48 × 10^5^ C + 0.345	3.81 × 10^–7^ to 1.90 × 10^–6^	5.06 × 10^–7^	1.68 × 10^–6^	*R*^2^ = 0.9965
AA	Δ*A* = 0.75 × 10^4^ C – 0.0004	4.76 × 10^–6^ to 5.71 × 10^–5^	3.24 × 10^–6^	1.08 × 10^–5^	*R*^2^ = 0.9901
EC	Δ*A* = 0.55 × 10^5^ C + 0.0977	9.52 × 10^–7^ to 4.76 × 10^–6^	4.42 × 10^–7^	1.47 × 10^–6^	*R*^2^ = 0.9932
FA	Δ*A* = 1.04 × 10^4^ C + 0.0816	3.81 × 10^–6^ to 1.90 × 10^–5^	2.34 × 10^–6^	7.79 × 10^–6^	*R*^2^ = 0.9935
CYS	Δ*A* = 0.16 × 10^4^ C + 0.0003	2.38 × 10^–5^ to 2.38 × 10^–4^	1.52 × 10^–5^	5.06 × 10^–5^	*R*^2^ = 0.9961
CFA	Δ*A* = 0.20 × 10^5^ C – 1.608	3.81 × 10^–6^ to 1.90 × 10^–5^	1.22 × 10^–8^	4.06 × 10^–8^	*R*^2^ = 0.9718
GSH	Δ*A* = 0.27 × 10^4^ C + 0.071	1.90 × 10^–5^ to 9.52 × 10^–5^	9.00 × 10^–6^	3.00 × 10^–5^	*R*^2^ = 0.9844
NAC	Δ*A* = 0.75 × 10^4^ C – 0.0285	9.52 × 10^–6^ to 4.76 × 10^–5^	3.24 × 10^–6^	1.86 × 10^–5^	*R*^2^ = 0.9889
QR	Δ*A* = 0.52 × 10^4^ C + 0.0495	4.76 × 10^–6^ to 4.76 × 10^–5^	4.67 × 10^–6^	1.55 × 10^–5^	*R*^2^ = 0.9805
NG	Δ*A* = 0.21 × 10^4^ C + 0.0673	1.90 × 10^–5^ to 9.52 × 10^–5^	1.16 × 10^–5^	3.58 × 10^–5^	*R*^2^ = 0.9871
p-CUM	Δ*A* = 0.17 × 10^4^ C + 0.0607	1.90 × 10^–5^ to 9.52 × 10^–5^	1.43 × 10^–5^	4.76 × 10^–5^	*R*^2^ = 0.9930

After the determination of radical scavenging efficiencies by means of the proposed method and the reference method, the rank order of AOx compounds for scavenging SORs was found and compared. A meaningful comparison between methods having different mechanisms could be made on the basis of 50% inhibitory concentrations (IC_50_ values) of the tested AOx, calculated *via* inhibition (scavenging) percentages defined below
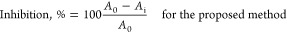
and
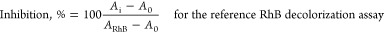


The 50% inhibitory concentrations (IC_50_) of AOx were found from the graphs of inhibition (%) against concentration, from which the IC_50_ value corresponded to the concentration yielding 50% radical scavenging. The calculated IC_50_ values with the two methods are tabulated in Table 4.

**Table 4:** IC_50_ Values of Tested AOx Compounds Calculated by the Proposed TMB Method and the Reference RhB Decolorization Assay

AOx	IC_50_ (TMB) (M)	IC_50_ (RhB) (M)
GA	5.60 × 10^–6^	2.50 × 10^–5^
CAT	1.06 × 10^–6^	1.14 × 10^–6^
AA	1.20 × 10^–5^	2.81 × 10^–5^
EC	6.06 × 10^–7^	1.08 × 10^–6^
FA	6.03 × 10^–6^	6.20 × 10^–6^
CYS	2.15 × 10^–5^	1.39 × 10^–4^
CFA	2.65 × 10^–6^	1.03 × 10^–5^
GSH	2.40 × 10^–5^	4.26 × 10^–5^
NAC	2.25 × 10^–5^	3.10 × 10^–5^
QR	2.85 × 10^–6^	2.80 × 10^–5^
NG	3.76 × 10^–5^	4.46 × 10^–5^
p-CUM	4.02 × 10^–5^	7.60 × 10^–5^

The SOR scavenging effects of AOx compounds were ranked according to the IC_50_ values, calculated for the TMB assay
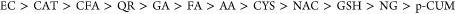
and for the RhB method
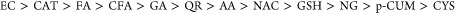


To examine the compatibility between the two methods, these two AOx efficiency orders were compared by using a statistical method—Spearman correlation coefficient.^13^ The correlation coefficient was found as
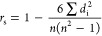
where *r*_s_ is Spearman’s rank correlation coefficient, *d*_i_ is the difference between paired ranks, and *n* is the number of pairs.

Accordingly, the *r*_s_ value was determined as 0.88, indicating a very strong correlation between the two methods used for investigating the SOR scavenging abilities of AOxs. It is particularly interesting that the leading three AOx compounds in both methods (EC, CAT, and CFA) ranked in the same places as the most powerful scavengers. It is known that there is a strong relationship between the AOx capacity and chemical structure for phenolic compounds. In particular, the number and position of –OH groups and the unsaturation of the ring with a conjugated structure are determinants on the activity of AOx.^31^ In addition, Bors *et al*. reported on flavonoids that the catechol structure in the B ring, the 2,3-double bond contributing to the electron delocalization of the same ring, and 3-and 5-hydroxyl groups increasing radical scavenging potential make CAT a highly effective AOx.^32^ Although quercetin fully bears these properties required by an ideal antioxidant, CAT proved to be a better scavenger than quercetin against SORs in our study. The higher electron-donating ability of quercetin than CAT brings superiority to quercetin in electron-transfer-based antioxidant capacity assays, but in our case, the inhibitive ability of transition metal ion-induced SOR generation is considered. *Ortho*-di/trihydroxy phenolics such as quercetin proved to be the most potent prooxidants among other phenolics, possibly due to their reducing power on transition metal ions to generate reactive species through Fenton-type reactions.^33^ It was previously demonstrated that dietary flavonols (particularly quercetin and myricetin) were able to produce ROS at physiological pH and in the presence of Fe.^34^ In a similar study, Ueda *et al*. examined hydroxyl radical scavenging activities of different AOx compounds by producing ROS from a combination of Cu(II)–ethylenediamine chelate and H_2_O_2_; the authors found that CAT could suppress DNA strand scission on a large scale, possibly *via* Cu–CAT complexation.^35^ Fe binding was found to be weaker for quercetin than for CAT, probably arising from the presence of conjugation extending from the C4-keto group, *via* C2–3 to the 3′-OH group (rings B and C), where the absence of conjugation (*via* the 4-keto group and the −3 alkene bond) appeared to enhance Fe binding at the 3′-OH and 4′-OH groups of CAT.^36^ A similar mechanism may be responsible in our case, involving relatively stable chelate formation between Co(II)—a borderline Lewis acid according to Pearson’s classification of hard/soft acids/bases and CAT –OH groups, being borderline Lewis bases, thereby suppressing SOR formation. The more Co(II) that is chelated by an antioxidant, the less SOR generation will be, depending on less availability of free Co(II) ions for sulfite activation. Under these circumstances, it is understandable that the proposed study shows CAT and EC as the most potent radical scavengers.

#### 3.3.3 Determination of Additivity Effect of the Binary and Ternary AOx Mixtures on Radical Scavenging

Binary and ternary mixtures of AOxs were prepared as given in Section 2.6. First of all, the proposed method was applied to each AOx compound, forming the mixture one by one, and then applied to the mixture. In order to calculate the differential absorbance (Δ*A*) values, the absorbance measured in the presence of AOx compound(s) was subtracted from that measured in the absence of AOx. The Δ*A* values found for individual AOx compounds were mathematically summed up and named as “Theoretical Δ*A*”. On the other hand, the absorbance of the binary (or ternary) mixture was also measured. The difference between measured absorbance in the presence and absence of AOx mixture was named as “Experimental Δ*A*”. Finally, the error (%) was calculated by using the equation given below
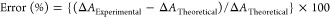


The error values calculated for the mixtures are collectively given in Table 5.

**Table 5:** Error (%) Values Calculated for Binary and Ternary Mixtures of the AOx Compounds

AOx mixture	error, %
GA + CFA	+5.67
GA + CYS	+2.56
CFA + CYS	–4.84
CAT + AA	+0.16
CAT + FA	–3.31
AA + FA	+5.18
GA + CFA + CYS	–7.70
CAT + FA + AA	+1.77

As can be seen from Table 5, all of the error (%) values were between +0.16 and −7.70, which can be accepted as satisfactory. Provided that the concentrations of AOx in a synthetic mixture are chosen to obey Beer’s law, obtaining additive results provides an understanding of true synergistic or antagonistic interactions for a real sample.^37^

### 3.4 Application of the Proposed Method to Real Samples for Determining SOR Scavenging Activity

Real sample experiments were performed as described in Section 2.8. It is known that the method used for extraction of phenolic contents of tea samples dramatically affects the results. For example, ISO methods suggest methanol extraction (aqueous solution at 70% concentration) at 70 °C.^38,39^ Nevertheless, we preferred a method used for tea consumption in daily life and used an infusion technique for extraction. Classically, while preparing tea as a regular hot beverage, ready-to-use tea bags are dipped into hot water by consumers. The results were calculated as mmol GA equivalent per gram tea sample and are presented in Table 6.

**Table 6:** SOR Scavenging Activity of Tea Samples in mmol GA equiv/g Tea Sample^a^

real sample	proposed TMB method (mmol GA/g)	RhB method (mmol GA/g)
white tea	0.837 ± 0.009	0.585 ± 0.011
green tea	0.349 ± 0.014	0.259 ± 0.015
black tea	0.168 ± 0.016	0.122 ± 0.017

a Results were given as  ± (*t*_0.95_. s/ ); *N* = 5 ( = mean, s = standard deviation).

To compare the precision of the proposed method with that of the reference RhB method, the *F* test was used. For this purpose, the *F* values were calculated for all of the tested tea samples separately. Accordingly, these values were found as 1.36 for white tea, 1.62 for green tea, and 1.17 for black tea, all smaller than the table *F* value (which was 6.39) corresponding to the respective degrees of freedom. The results indicated that the precision of the proposed method was not significantly different from that of the reference RhB method within the 95% confidence interval.

As can be seen from Table 6, the results were acceptably compatible, and the difference between the two set of results can be explained by the different working mechanisms and calculation methods. On the other hand, both methods concurred that among the tested real samples, white tea was the most effective SOR scavenger. The order of the radical scavenging effect of tested tea samples was determined as white tea > green tea > black tea. It is not surprising because white tea is only lightly fermented and processed so as to bear more nutrients than black and green teas.^40^ In fact, white tea is usually just dried and not fermented during processing.^41^ The fermentation process is expected to result in a reduction in the total content of CAT species in highly fermented, heat-treated teas. All three types of tea were obtained from *C. sinensis,* especially from its buds and leaves, the differences being caused by the genotype of the plant, growing techniques, and postharvest processing. Among the phenolic contents of tea samples, CATs are of special importance, making up about 30% of the dry weight of the tea leaves. Green tea was shown to have a higher CAT content than black tea.^42^ It is known that there is a strong relationship between the chemical composition of infused teas and their benefits on human health.^43^ Another important point is to decide on the unit for reporting experimental results, where the use of GA equivalent is usually acceptable.^41^ Therefore, in the presented study, the results were given as millimolar GA equivalent. In accordance with the lesson learned in this study, CAT and its derivatives are highly effective for SOR scavenging, and the tea plant rich in CATs is a potent scavenger. Additionally, different teas are consumed as infusions with a high CAT content. So, it can be concluded that tea consumption is a good way to cope with the harmful effects of SORs in the human body.

## 4 Conclusions

Certain transition metal ions are essential elements taking part in enzymatic reactions, but they may also catalyze the oxidation of sulfite entering the body from various sources. It is known that Co(II) is one of the most effective transition metal ions in this regard, capable of forming SORs with sulfite. The sulfate radical is one of the strongest oxidants which can cause damage to biomacromolecules, especially DNA. Therefore, in the proposed study, a simple and low-cost colorimetric method for determining SORs and simultaneously testing the radical scavenging activity of a number of AOx compounds was established. The determination of SOR scavengers is also important from the standpoint of water treatment processes because they may hinder radicalic degradation of contaminants in treated water. For this purpose, a very well-known colorimetric reagent, that is, TMB, was used. Although TMB is not very selective toward SORs, the method of generation of these radicals (*i.e*., Co(II) combined with sulfite) was selective, and other ROS could be excluded from the system by proper solvent selection. The obtained results were compared with those of the RhB decolorization assay, and consistent results were achieved. The proposed assay was more sensitive than the RhB method in determining the SOR scavenging activity because it yielded higher molar absorptivities and lower detection limits for AOxs. Among the tested AOx compounds, CAT and EC were determined as the most effective for SOR scavenging. In addition, white, green, and black tea infusions were tested as real samples, yielding the order of effectiveness on radical scavenging as white > green > black tea. The results of this study are expected to serve further research on the detection and scavenging of SOR in biochemistry, food chemistry, and environmental chemistry.
